# Malpractice Claims and Incident Reporting: Two Faces of the Same Coin?

**DOI:** 10.3390/ijerph192316253

**Published:** 2022-12-05

**Authors:** Giuseppe Vetrugno, Federica Foti, Vincenzo M. Grassi, Fabio De-Giorgio, Andrea Cambieri, Renato Ghisellini, Francesco Clemente, Luca Marchese, Giuseppe Sabatelli, Giuseppe Delogu, Paola Frati, Vittorio Fineschi

**Affiliations:** 1 UOS Risk Management Fondazione Policlinico A. Gemelli IRCCS, Department of Health Surveillance and Bioethics, Section of Legal Medicine, School of Medicine, Università Cattolica del Sacro Cuore, L.go F. Vito 1, 00168 Rome, Italy; 2 Fondazione Policlinico A. Gemelli IRCCS, L.go A. Gemelli 8, 00168 Rome, Italy; 3 Strategica Risk Consulting Srl, 20123 Milano, Italy; 4 Responsabile Centro Regionale Rischio Clinico Regione Lazio, 00145 Rome, Italy; 5 Department of Anatomical, Histological, Forensic and Orthopedic Sciences, Sapienza University of Rome, 00128 Rome, Italy

**Keywords:** malpractice claims, incident reporting, risk management

## Abstract

Incident reporting is an important method to identify risks because learning from the reports is crucial in developing and implementing effective improvements. A medical malpractice claims analysis is an important tool in any case. Both incident reports and claims show cases of damage caused to patients, despite incident reporting comprising near misses, cases where no event occurred and no-harm events. We therefore compare the two worlds to assess whether they are similar or definitively different. From 1 January 2014 to 31 December 2021, the claims database of Policlinico Universitario A. Gemelli IRCCS collected 843 claims. From 1 January 2020 to 31 December 2021, the incident-reporting database collected 1919 events. In order to compare the two, we used IBNR calculation, usually adopted by the insurance industry to determine loss to a company and to evaluate the real number of adverse events that occurred. Indeed, the number of reported adverse events almost overlapped with the total number of events, which is indicative that incurred-but-not-reported events are practically irrelevant. The distribution of damage events reported as claims in the period from 1 January 2020 to 31 December 2021 and related to incidents that occurred in the months of the same period, grouped by quarter, was then compared with the distribution of damage events reported as adverse events and sentinel events in the same period, grouped by quarter. The analysis of the claims database showed that the claims trend is slightly decreasing. However, the analysis of the reports database showed that, in the period 2020–2021, the reports trend was increasing. In our study, the comparison of the two, malpractice claims and incident reporting, documented many differences and weak areas of overlap. Nevertheless, this contribution represents the first attempt to compare the two and new studies focusing on single types of adverse events are, therefore, desirable.

## 1. Introduction

In 2018, *The Economist* published an article about the organizational models adopted by hospitals to reduce the incidence of medical errors and created a sense of alarm by estimating the number of patients who have been victims of avoidable adverse events with harmful *consequences* [1]. Twenty years have passed since the start of the campaign “To err is human” [2], and avoidable medical damage still constitutes the “14th leading cause of ill health globally—a burden akin to malaria” [1], raising doubts about the success of the efforts made to date to ensure patient safety. We wondered if these twenty years resemble those depicted in Horace’s famous joke in *Ars Poetica*: “Parturient montes, nascetur ridiculus mus” (i.e., “The mountain labours and brings forth a little mouse”) [3].

Clinical risk management (CRM) is an organizational response that aims to improve the quality and safety of healthcare services by identifying the risks of damage to patients’ health and acting to prevent them. CRM is usually a four-step process, where the risk is first identified and then analyzed in terms of its root causes; the results of the analysis guide actions intended to reduce the risk. The effectiveness of these actions is finally assessed. Incident reporting is an important method to identify risks because learning from the reports is crucial in developing and implementing effective improvements [4].

Incident reporting is an operator-dependent tool and, therefore, may be affected by insufficient descriptive power or poor data input due to the scarce compliance of health professionals. Therefore, the use of alternative tools is growing, i.e., the use of artificial intelligence (A.I.) for the extraction of adverse events and near misses directly from medical records [5].

These instruments have shown some limitations; as already reported in the literature regarding examples of A.I. in the medical field, algorithms may suffer from the poor reproducibility of the data set on which they were built and tested [6,7]. Moreover, although electronic medical records are now widely used, digitalization is not yet complete; this is why adverse events, such as falls, may not be noted, thus escaping A.I. interception [8]. Finally, to date, the method used to calculate the sensitivity and specificity of A.I. tools in searching for adverse events throughout medical records is not well defined [9], and the limits of a pure analysis of big data have been demonstrated since large databases can result in spurious correlations [10].

Based on these observations, the classic tool of incident reporting still remains valid and reliable; this is also because it indirectly provides data on the diffusion of the culture of safety among healthcare workers and about the full realization of the no-blame culture by health organizations [4].

A medical malpractice claims analysis is another important tool in any case [11]. Both incident reports and claims show cases where damage to patients occurred, despite incident reporting comprising near misses, cases where no event occurred and no-harm events. 

Indeed, observing a claim is similar to observing a star; the light comes from an object that may no longer exist. In the same way, the knowledge of what went wrong (the reason for the claim) may not be useful for any CRM policy, because it refers to a different historical time and organizational context. In contrast, incident reporting resembles a lamp that lights up on the dashboard of a moving machine and suggests that one must stop to analyze what happened and why. Apparently, the respective “lights” of claims and adverse events reflect two different worlds.

After all, the techniques of the analyses applied to both worlds are also different. In the claims contest, the evaluation is focused on the search for the culprit, and this is not the case in incident reporting. Moreover, in the insurance industry and, therefore, in claims management, it is common practice to punctually determine the events that certainly happened but have not yet been reported, the so-called incurred-but-not-reported (IBNR) claims, which are usually estimated using actuarial instruments for economic purposes [12,13,14]. This does not happen in the incident-reporting world. Classically, the IBNR calculation is based on the “chain ladder” method and IBNR claims are calculated by multiplying the previous year’s IBNR claims amount by the ratio of change in the incurred losses amount [15]. The “chain ladder” method is thoroughly explained in Appendix A.

To compare the two data sets, we assumed that the IBNR calculation should also be applied to incident reporting; in fact, it could be that adverse events that took place have not yet been reported, or their consequences remain unknown to health professionals, because the damage occurred far from the incidents.

So, are the two worlds truly different, as observed above? Or might there be a relationship between them? 

In this study, to the best of our knowledge, the IBNR calculation is applied to the world of incident reporting for the first time; in this way, a data comparison may potentially allow for the above questions to be answered.

## 2. Materials and Methods

Our teaching hospital, Policlinico Universitario A. Gemelli IRCCS, is one of the largest hospitals in Rome, with 48 operational units, 1538 beds, 53,765 patients who recovered in 2021, and records of more than 80,000 admissions to the emergency room per year in the pre-COVID-19 era (64,069 in 2021); moreover, it provides hundreds of thousands of outpatient services annually. In this health organization, since June 2018, special software has been collecting reports of near misses, no-harm events, adverse events and sentinel events (the most severe adverse events), eventually recording root cause analyses and auditing and assessing the corrective actions for each report. Other types of reports are those related to falls, injuries and violence against staff. 

Moreover, our risk management unit has a database of medical malpractice claims; those received from January 2014 to March 2022 were taken into account and a matrix describing the months of occurrence of the damage events, ordered by months of request, was built. On this basis, IBNR claims were estimated through the so-called chain-ladder method [16] (Appendix A and Supplementary Material Tables S1 and S2).

The fundamental hypothesis of such a method assumes that a historical view of the development patterns is indicative of their future trend. The actuarial valuation of the matrix shows the temporal trend of the claims-related damage events that occurred in the period 2014–2021 and were reported until 31 March 2022. The matrix is therefore constructed by taking into account the claims that arrived within the first quarter of 2022, but it refers to events that occurred until 31 December 2021 in order to make the claim accounts more reliable. 

The same matrix was built using the database of incident reports collected monthly in the period from 1 January 2020 to 31 December 2021. The rationale was the following: the software for compiling and sending incident reports was introduced on 1 June 2018 (previously, incident reports were compiled on paper reports and amounted to a few hundred reports per year) and only produced reliable results from 1 January 2020, taking advantage of the Joint Commission International (JCI) accreditation standards [4]. Since then, incident reporting has spread and reporting quality has improved.

In addition, in order to make the reports comparable to the claims, all near misses and no-harm events were removed from the matrix, as well as the adverse events involving healthcare workers, such as violence and injuries.

On this basis, adverse events that took place but were not reported (similarly to IBNR claims) were estimated through the so-called chain-ladder method. The actuarial valuation of this matrix shows the temporal trend of damage events collected by the incident-reporting system.

Finally, the relative variability observed between the two series was calculated using R-squared (R^2^) (this value, which ranges from 0 to 1, indicates to what extent the variance of the first variable explains the variance of the second variable). Moreover, we calculated Pearson and Kendall correlation coefficients.

## 3. Results

The claims database of Policlinico Universitario A. Gemelli IRCCS collected 843 claims related to events that occurred in the period from 1 January 2014 to 31 December 2021. We only considered claims concerning harmful and negative outcomes for patients, thus excluding refund requests for lost personal items or similar cases; thus, the so-structured database consisted of 481 claims, defined as “proper claims”. 

Claims database is shown in Table 1.

From 1 January 2020 to 31 December 2021, the incident-reporting database collected 1919 events.

Table 2 summarizes the incident-reporting database.

The claims matrix highlights a prolonged temporal delay in notification: an event suffered by a patient can be reported as a claim several months/years from the time of occurrence (see Supplementary Material Table S1). 

**Figure 1:**
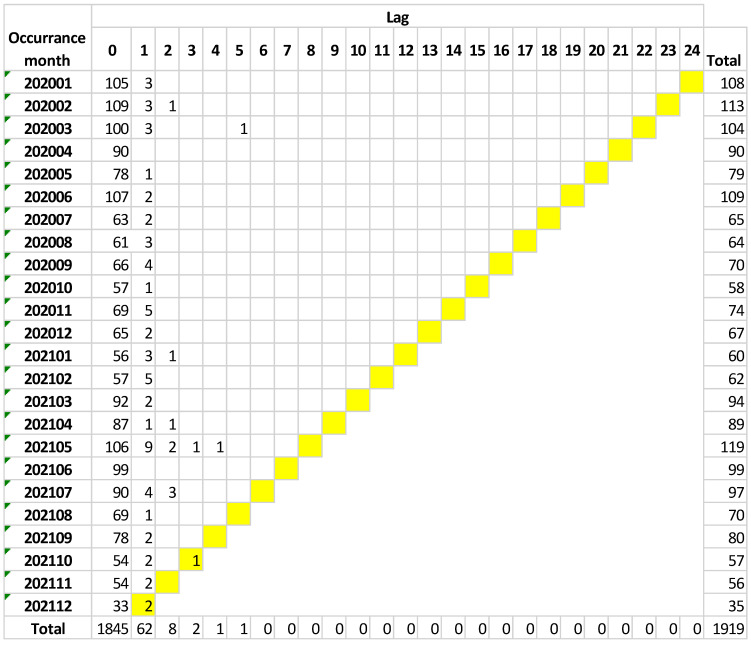
Temporal latency trend related to the occurrence of adverse events.

On the contrary, in the incident-reporting system, the temporal latency is absent or limited to one month after the occurrence of the event (rarely four months from the date of occurrence), as shown in Figure 1.

The analysis of the incident-reporting databases showed that healthcare workers do not report events after 4–5 months have passed from the date of occurrence.

**Figure 2:**
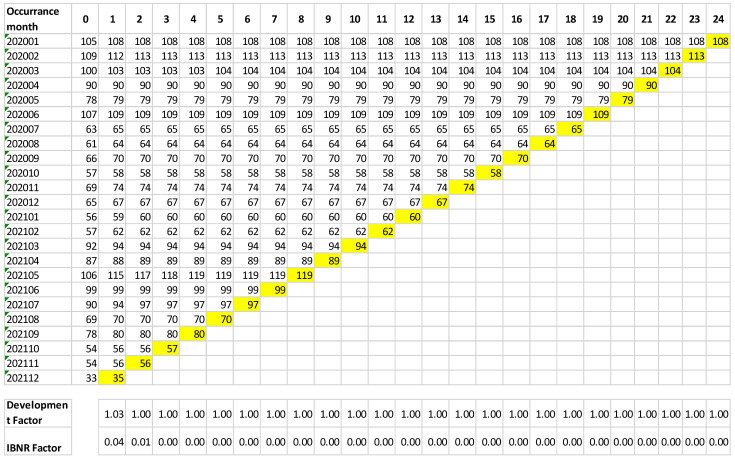
Incurred but not reported evaluation distributed by timing.

The IBNR calculation revealed that the predictive power of IBNR adverse events is almost null in the incident-reporting database, as shown in Figure 2.

In order to further reduce the variability related to monthly characteristics (i.e., holidays), merging per quarter was chosen (Table 3).

**Figure 3:**
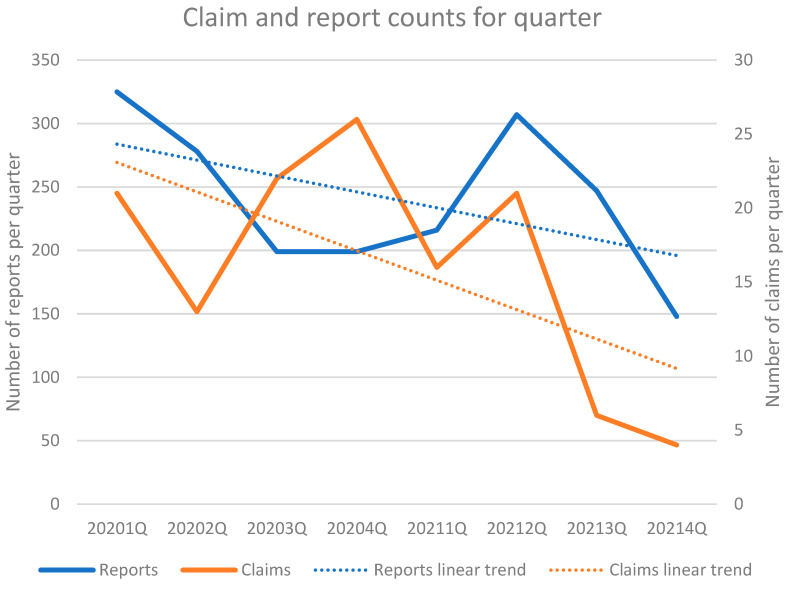
Claim and report counts (years 2020–2021) for quarter.

The analysis of the claims database showed that, in the period 2014–2021, the claims trend was slightly decreasing. The same trend was also observed in the period limited to 2020–2021 when compared to the incident-reporting database, which, instead, showed an increasing trend, as well as when analyzed per quarter (Figure 3).

**Figure 4:**
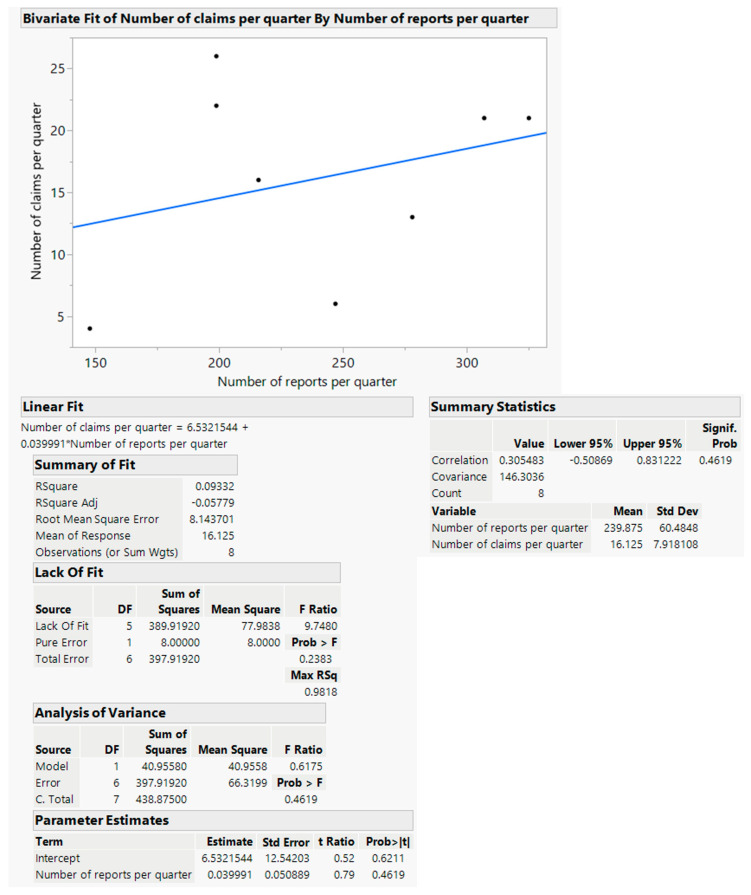
Number of claims versus number of reports.

**Figure 5:**
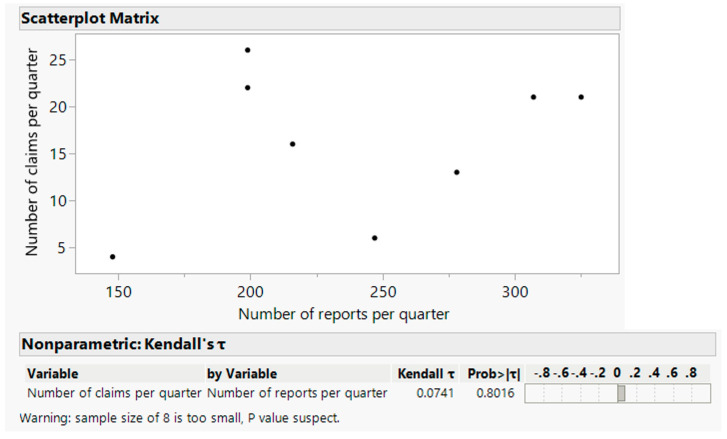
Kendall rank correlation.

The distribution of claims in the period of 2020–2021, grouped by quarter, was compared with the distribution of incident reporting in the same period, grouped by quarter, using JMP statistical software. This comparison, shown in Figure 4, reveals a Pearson linear correlation coefficient R of 0.31, with a high *p*-value of 0.46, and a determination coefficient R^2^ of 0.093, with high *p*-values of 0.62 and 0.46 for the intercept and slope coefficients of the linear fitting, respectively. In Figure 5, the analysis of the Kendall rank correlation coefficient gives a low positive value of the τ coefficient of 0.0741, with a high *p*-value of 0.8016.

## 4. Discussion

Incident reporting is one the most important tools for risk stratification, as well as for health organizations that want to ensure patient safety. That is, the reports are the starting point of the “learning from mistakes” process in a ‘no blame’ context, where the focus is on the root causes of the mistake, often related to organizational factors. Only if healthcare workers and other staff members are aware of this context will they use the incident-reporting system properly. Incident reporting, therefore, is required by the most important accreditation institutions, such as the JCI. There is another reason that supports the requirement of incident reporting: only an immediate knowledge of the occurrence of an adverse event allows for non-conflictual management of the patient and their family members [17].

In our study, the comparison of the two worlds, malpractice claims and incident reporting, documented many differences and weak areas of overlap.

The first difference is the trend: the incident-reporting database has a growing trend, while the claims database has a decreasing trend. This trend could also be explained by changes that occurred due to the COVID-19 pandemic [18,19], with reallocation of internal space, redistribution of resources and, predominantly, downward remodulation of elective procedures.

The observed increase in the reporting trend and the quality improvement suggests the progressive spread of incident reporting, even if some papers highlighted possible overestimations, disappointments and shortcomings of incident reporting, leading to contaminated data [20,21,22,23].

In the claims world, we observed a decrease in the years from 2014 to 2021. This could suggest an improved quality of the assistance provided during that period of time, which the matrix and IBNR evaluation seems to confirm. Similarly, the same decrease was observed in the USA in the period from 1992 to 2014, when “the rate of paid claims decreased by 55.7% (from 20.1 to 8.9 per 1000 physician-years)” [24]. However, a risk manager should not believe that this means that the risk management policies adopted over the years were effective: instead, it could suggest that the intention of the injured patient to initiate litigation has weakened over time because, for example, the proactive approach and communication policies have improved over time [25].

A second difference concerns the results observed following the application of the IBNR calculation to the two worlds. In the case of malpractice claims, the long-standing instrument makes it possible to calculate the actual number of events occurring over a given period of time and, thus, to guide policies for loss provision. The estimate of IBNR claims is very useful in the case of claims because the latency time for claims could be very long; in Italy, claims could arrive up to 10 years after the event took place; in our database, the maximum interval was 7 years.

In the world of incident reporting, the IBNR calculation does not affect the total number of events. This is related to the very short latency time between the event and its reporting: 99% of events are reported within the first week after their occurrence and only 0.01% of events are reported later, particularly after 5 months of their occurrence. 

This diversity turns into a third difference, which affects the use of a single data source: claims involve a delay that makes it difficult to extract useful indications from an in-depth analysis of the claim and its causes. A medical malpractice data set is already considered a valuable resource that can show the effectiveness of safety policies adopted by health organizations via an analysis of adverse events that have occurred [11,26]. Nevertheless, temporal latency makes it barely functional for safety purposes: health organizations evolve over the course of a few months, so an event may not be more significant if analyzed many months or even years after its occurrence. Incident reporting, however, provides a more immediate contribution, polarized in the search of causes not strictly operator-dependent but rooted in the organization by means of the recent memory of the operator and their point of view.

However, it must be noted that some have suggested the introduction of the same tools used for actuarial assessment specific of the insurance sector to improve the selection of healthcare workers based on their risk of producing claims [27]. It is known that, especially in areas where it is tough to find adequate insurance cover, “physicians with multiple malpractice claims were no more likely to relocate geographically than those with no claims, but they were more likely to stop practicing medicine or switch to smaller practice settings” [28]. However, such an approach is far from the ‘no-blame’ logic and forgets the assumption that, when an adverse event occurs, “healthcare professionals have fought a battle and lost” [29].

The descriptive differences so far seem to be confirmed by the statistical evidence that emerged in our study, indicating that there is no correlation between the two worlds.

In particular, the linear Pearson correlation appeared as a weak correlation with a low statistical significance (a low R coefficient and a high *p*-value). Linear fitting showed a poor level for both the capability of explaining the variability and the statistical significance of the fitting parameters (a low R^2^ coefficient and high *p*-values). A simple visual inspection of the data also excluded non-linear relationship evidence. The sign of the τ Kendall coefficient was coherent with the sign of the Pearson coefficient R, but its statistical significance was low (high *p*-value).

Despite these data, however, we cannot overlook the weight of some of the common elements that the statistical validity of this study was not able to express. 

There is no doubt that the damage suffered by a patient is the same in both areas. The difference is the voice that describes it and the purposes that move the reports. In the claims world, the damage is reported by the patients (or their lawyer); in the incident-reporting world, however, the event is described by the operators. Claims come from the feeling that medical errors have overturned patients’ or their relatives’ lives; incident reports instead come from healthcare workers who care about enhancing quality and safety.

There is also no doubt that gruesome events may not be reported by operators due to shame, but they become immediately known when a malpractice claim arrives.

Bolcato et al. suggested that a claim analysis can enhance the awareness of sentinel events that were not reported by healthcare workers due to a variety of factors [30].

Many claims stem from adverse events, and many adverse events give rise to claims. A cause-and-effect relationship exists, but it only explains a minority of the cases, as already stated [31] and as suggested by the low R^2^ coefficient that we found.

Many factors play a role in giving rise to a claim, including a delay in diagnosis or treatment, unexpected events (such as surgical complications), patient and family expectations that are far from scientific possibility, information that is not as clear as expected, and a patient–practitioner relationship that is not as satisfying as expected. Therefore, not every claim comes from an adverse event. An adverse event is an undesirable occurrence in patient management and does not necessarily mean that the iatrogenic damage is related to malpractice. Adverse events occur for many different reasons, such as patient-related factors (e.g., wrong identification) and system-related factors (the root causes investigated in CRM).

Our data suggest that, as Bolcato et al. stated [30], an analysis of claims could be an important tool for risk management, providing a different point of view on the quality of care and different types of information.

For example, in our healthcare organization in 2021, some falls were not reported, even if they occurred in the Emergency Department (whose staff is highly trained on incident reporting) and caused notable damage to patients, but they gave rise to claims. These events become known after the arrival of related claims with a four-month delay.

This means that, if there had not been integration between the two databases, the knowledge of the falls in that exact setting of care would have been partial, and, therefore, the quality could have been affected, because the root causes would not have been investigated; instead, claims generated improvement actions.

Other cases not presented in the incident-reporting database but recorded in the malpractice claims database do not offer the same possibilities for immediate improvement, but they can provide an opportunity to question the resilience and effectiveness of the patient safety measures adopted over the years by the health organization. In our experience, some cases in the malpractice database concern bowel perforations that occur during laparoscopy: these events are not present in the incident-reporting database. This can be explained by the fact that perforation is a known surgical complication, and, thus, it is not considered an adverse event by surgeons. Two claims regarded retained surgical bodies in the abdomen, but the surgeons did not notice this during the procedure (the patients found out during surgical procedures that occurred later in other hospitals) and were notified three and five years after the event. Therefore, these claims, which did not arise from incident reporting, evaluated on the basis of scientific methods that guarantee objectivity, reproducibility and rigor [32], could represent organizational failures that healthcare professionals do not notice and could provide information on patient satisfaction.

## 5. Conclusions

The results of this study do not prove that a specific insurance tool (IBNR) could be useful in risk prevention. We used IBNR calculation, usually adopted by the insurance industry to determine the loss of a company, in the evaluation of the real number of adverse events reported to the risk management unit. However, the number of reported adverse events almost overlapped with the total number of events, which is indicative that incurred-but-not-reported events are practically irrelevant. A probable reason for this finding is the use of the entire database of adverse events. As already suggested [33], other insurance tools could be usefully applied in risk prevention, considering that some analytical methods of Clinical Risk Management (e.g., Root Cause Analysis) have approaches based on the reconstruction of causal links and the identification of contributing factors for the reconstruction of liability chains, similar to those already used in the insurance industry. Likewise, building a database of specific adverse events (e.g., falls) by the use of techniques specific to insurance science, a health facility could develop a predictive model. These databases and the predictive models would help to foresee how many events of that specific category could occur in the future, with a certain frequency, and thus to understand if the procedures adopted to contain the phenomenon are functioning or not. New studies focusing on single types of adverse events (for example, falls) are, therefore, required [34].

## Figures and Tables

**Table 1:** Malpractice claims database presented by the month of occurrence; total number of claims (used to estimate the number of IBNR claims); number of proper claims; IBNR factor (obtained by the successive multiplication of the progression index); estimated number of IBNR claims (the proper claim number multiplied by the IBNR factor); sum of proper claims and IBNR claims.

Month of Occurrence	Number of Claims	Number of Proper Claims	IBNR Factor	Number of IBNR Claims Rounded to Units	Number of Proper Claims + IBNR Claims
201401	18	7	0	0	7
201402	12	6	0	0	6
201403	18	8	0	0	8
201404	12	6	0	0	6
201405	12	5	0	0	5
201406	16	10	0	0	10
201407	11	4	0	0	4
201408	11	4	0	0	4
201409	11	8	0	0	8
201410	14	7	0	0	7
201411	17	6	0	0	6
201412	11	4	0	0	4
201501	14	7	0	0	7
201502	10	4	0	0	4
201503	7	4	0	0	4
201504	17	7	0	0	7
201505	9	3	0	0	3
201506	14	5	0.00457	0	5
201507	14	9	0.00457	0	9
201508	12	5	0.00457	0	5
201509	12	6	0.00457	0	6
201510	3	0	0.00457	0	0
201511	12	6	0.00457	0	6
201512	13	6	0.00457	0	6
201601	15	8	0.00457	0	8
201602	17	14	0.00457	0	14
201603	11	6	0.00761	0	6
201604	11	5	0.01057	0	5
201605	14	6	0.01057	0	6
201606	10	3	0.01335	0	3
201607	13	7	0.01607	0	7
201608	8	3	0.01607	0	3
201609	9	7	0.01865	0	7
201610	4	3	0.01865	0	3
201611	7	5	0.02117	0	5
201612	11	3	0.02117	0	3
201701	10	7	0.02359	0	7
201702	10	8	0.02359	0	8
201703	10	6	0.02591	0	6
201704	9	4	0.05636	0	4
201705	13	6	0.09051	1	7
201706	8	7	0.09542	1	8
201707	9	4	0.10274	0	4
201708	3	1	0.11246	0	1
201709	8	7	0.11735	1	8
201710	4	2	0.12464	0	2
201711	7	3	0.13442	0	3
201712	7	6	0.14175	1	7
201801	10	2	0.154	0	2
201802	8	6	0.16378	1	7
201803	13	11	0.16378	2	13
201804	10	4	0.1733	1	5
201805	10	7	0.17802	1	8
201806	8	6	0.18976	1	7
201807	12	10	0.20392	2	12
201808	5	3	0.2086	1	4
201809	8	5	0.21328	1	6
201810	9	7	0.22259	2	9
201811	12	11	0.22955	3	14
201812	6	5	0.24107	1	6
201901	11	7	0.24337	2	9
201902	11	6	0.24791	1	7
201903	12	8	0.26145	2	10
201904	8	5	0.27727	1	6
201905	6	3	0.27953	1	4
201906	8	4	0.29312	1	5
201907	5	5	0.30911	2	7
201908	5	4	0.33722	1	5
201909	6	5	0.35646	2	7
201910	9	8	0.36374	3	11
201911	5	3	0.37341	1	4
201912	5	5	0.38804	2	7
202001	7	6	0.39789	2	8
202002	8	6	0.41775	3	9
202003	4	3	0.43535	1	4
202004	7	3	0.46897	1	4
202005	4	2	0.49018	1	3
202006	7	4	0.51737	2	6
202007	5	4	0.54522	2	6
202008	3	3	0.58854	2	5
202009	9	7	0.62478	4	11
202010	10	6	0.65889	4	10
202011	6	3	0.72344	2	5
202012	9	6	0.80319	5	11
202101	5	3	0.86588	3	6
202102	5	3	0.957	3	6
202103	5	2	1.05183	2	4
202104	3	3	1.17503	4	7
202105	7	6	1.30173	8	14
202106	1	0	1.44159	0	0
202107	2	0	1.59911	0	0
202108	3	1	1.80944	2	3
202109	1	1	2.00765	2	3
202110	0	0	2.24588	0	0
202111	1	1	2.58942	3	4
202112	1	0	3.14744	0	0

**Table 2:** Incident-reporting database ordered by month of occurrence; total number of reports (used to estimate the number of IBNR events); IBNR factor (obtained with successive multiplications of the progression index); estimated number of IBNR events (the reported number multiplied by the IBNR factor); sum of the number of reports and IBNR events.

Month of Occurrence	Number of Reports	IBNR Factor	Number of IBNR Events Rounded to Units	Number of Reports + IBNR Events
202001	108	0	0	108
202002	113	0	0	113
202003	104	0	0	104
202004	90	0	0	90
202005	79	0	0	79
202006	109	0	0	109
202007	65	0	0	65
202008	64	0	0	64
202009	70	0	0	70
202010	58	0	0	58
202011	74	0	0	74
202012	67	0	0	67
202101	60	0	0	60
202102	62	0	0	62
202103	94	0	0	94
202104	89	0	0	89
202105	119	0	0	119
202106	99	0	0	99
202107	97	0	0	97
202108	70	0	0	70
202109	80	0.00059	0	80
202110	57	0.00116	0	57
202111	56	0.00226	0	56
202112	35	0.00654	0	35

**Table 3:** Temporal latency trend related to the occurrence of the event.

Quarter Distribution	Number of Reports	Number of Claims
2020-1Q	325	21
2020-2Q	278	13
2020-3Q	199	22
2020-4Q	199	26
2021-1Q	216	16
2021-2Q	307	21
2021-3Q	247	6
2021-4Q	148	4

## Data Availability

Data are available upon reasonable request.

## Supplementary Materials

The following supporting information can be downloaded at: https://www.mdpi.com/article/10.3390/ijerph192316253/s1, Table S1: Triangular two-dimensional matrix showing which divided claims occurred in the same month (rows), and which notification arrived in the same month as the event, one month later, two months later, and so on (columns); Table S2: Run-off matrix.

## Appendix A. The Chain-Ladder Method

The chain-ladder method comprises the following steps:

1. Let M be the two-dimensional triangular matrix, where rows M^i^, i = 1, …, p correspond to the month of the occurrence of the claim (usually ascending from the top row M^1^ to the bottom row M^p^), and columns Mj, j = 0, …, d, with d ≤ p − 1, represent the time unit (month) of delay in the claim process (usually from left to right). Thus, the two-dimensional matrix values of each cell Mj^i^ represent the number of claims that occurred in month i and were identified during month i + j, with i + j ≤ p. That is, the first column includes claims for compensation arriving in the month of the event, the second column shows those whose requests arrived one month later etc.
2. Let D be the triangular matrix (run-off matrix), where rows D^i^ and columns Dj have the same meaning as those in matrix M, but values Dj^i^ are the cumulative values given by the total amount of claims as the sum:

$$D_{j}^{i}:=\overset{j}{\underset{k=0}{\sum }}M_{k}^{i}$$

For each couple of successive columns D_j_ and D_j+1_, let R_j+1,j_ be the development ratio:

$$R_{j+1,j}:=\frac{\sum_{k=1}^{p-j-1}D_{j+1}^{k}}{\sum_{k=1}^{p-j-1}D_{j}^{k}}\geq 1$$

The product F(t), defined as

$$F\left(t\right):=\overset{t-1}{\underset{j=0}{\prod }}R_{j+1,j},t=1,\ldots ,d$$

represents the “average” progression of the cumulative claim, counting along successive “discovery” months.

For each month of occurrence (rows in the matrix), the fully developed number of claims is estimated by multiplying the cumulative number of claims determined so far (time t − 1) by the development factor *DF*(*t*), defined as

$$DF\left(t\right):=\{\begin{array}{c} F\left(d\right)\\ F\left(d\right)/F\left(t\right) \end{array}\begin{array}{c} t=1\\ t=2,\ldots ,d \end{array}$$

This method is based on a two-dimensional matrix, which counts, within a known time frame, the number of claims along the two temporal dimensions that define the rows and columns. The rows, in their sequence from top to bottom, represent the succession of the months of the occurrence of the claims. The columns, instead, indicate the interval of time, expressed in months, between the month of the occurrence of each event and the month of the arrival of the claim. This time interval increases from left to right, with respect to the succession of the columns. The count contained in each cell then represents the number of claims that occurred in the month corresponding to the row and the number of claims notified after the period expressed in months reported in the column (Supplementary Material Table S1). The cumulative values per line of these counts represent the progression of the arrival of the claims. The value obtained by adding the value of 1 to that of the average increase in the cumulative counts, calculated for all pairs of successive columns, constitutes the index of progression over time (development factor) of the number of claims. The calculated progression index is then applied, per row, to the number of claims determined so far in order to obtain an estimate of the total number of expected claims, consistent with the historical progression of the received claims. Using the progression index, the IBNR factor can be calculated by multiplying the successive values of the development index (see the run-off matrix—Supplementary Material Table S2).
